# Assessing the Underestimation of HIV Risk Infection among Young Men Who Have Sex with Men in Argentina

**DOI:** 10.3390/ijerph192215269

**Published:** 2022-11-18

**Authors:** Maria Feijoo-Cid, María Isabel Fernández-Cano, Virginia Zalazar, David Moriña-Soler, Rosa García-Sierra, Antonia Arreciado Marañón, Omar Sued

**Affiliations:** 1 Department of Nursing, Faculty of Medicine, Universitat Autónoma de Barcelona, 08193 Bellaterra, Spain; 2 Grup de REcerca Multidisciplinar en SAlut i Societat (GREMSAS), (2017 SGR 917), 08007 Barcelona, Spain; 3 Dirección de Investigaciones, Fundación Huésped, Buenos Aires C1202ABB, Argentina; 4 Department of Econometrics, Statistics and Applied Economics, Universitat de Barcelona, Riskcenter-IREA, 08035 Barcelona, Spain; 5 Research Support Unit Metropolitana Nord, Primary Care Research Institut Jordi Gol (IDIAPJGol), 08303 Barcelona, Spain

**Keywords:** HIV risk perception, HIV risk infection, men who have sex with men, adolescents, young adults

## Abstract

The aim of this study is to describe the discordance between the self-perceived risk and actual risk of HIV among young men who have sex with men (YMSM) and its associated factors. An online, cross-sectional study was conducted with 405 men recruited from an Argentinian NGO in 2017. Risk discordance (RD) was defined as the expression of the underestimation of risk, that is, as a lower self-perception of HIV risk, as measured with the Perceived Risk of HIV Scale, than the current risk of HIV infection, as measured by the HIV Incidence Risk Index. Multivariate logistic regression models were used to analyze the associations between the RD and the explanatory variables. High HIV risk was detected in 251 (62%), while 106 (26.2%) showed high self-perceived risk. RD was found in 230 (56.8%) YMSM. The predictors that increased RD were consistent condom use with casual partners (aOR = 3.8 [CI 95:1.5–11.0]), the use of Growler to meet partners (aOR = 10.38 [CI 95:161–121.94]), frequenting gay bars (aOR = 1.9 [95% CI:1.1–3.5]) and using LSD (aOR = 5.44 [CI 95:1.32–30.29]). Underestimation of HIV risk in YMSM is associated with standard HIV risk behavior and modulated by psychosocial aspects. Thus, prevention campaigns aimed at YMSM should include these factors, even though clinical practice does not. Health professionals should reconsider adapting their instruments to measure the risk of HIV in YMSM. It is unknown what score should be used for targeting high-risk YMSM, so more research is needed to fill this gap. Further research is needed to assess what score should be used for targeting high-risk in YMSM.

## 1. Introduction

In Latin America, the number of new HIV diagnoses increased by 21% between 2010 and 2019. It is estimated that 136,000 people in Argentina are HIV-positive, 17% of whom are unaware of their infection. One-third of the diagnoses are made in the advanced stages of the disease. The prevalence of HIV among men who have sex with men (MSM) is 12–15%. Among young people aged 15–24, more than 75% of the HIV cases are related to condomless anal intercourse with men [1,2]. In Chile, for instance, the number of new HIV infections has increased by 34% since 2010, and cases are specifically concentrated among young men who have sex with men (YMSM) [3]. A recent systematic review points to YMSM as possibly more vulnerable to HIV infection than older MSM in Latin America [4]. Latin America was once a region with great success in the deployment of HIV treatment programs but has lost momentum, allowing the epidemic to rebound among YMSM [3].

In the United States, HIV infections among YMSM aged 13–24 declined by 33% overall from 2015 to 2019, dropping in YMSM of all races. However, young Black/African American and Hispanic/Latino MSM continue to be severely and disproportionately affected [5]. Moreover, in the USA, only 56% of young people living with HIV are aware of being infected, the lowest rate of any age group [6].

Beliefs about the personal risk of HIV infection are key to understanding what might motivate people to engage in HIV risk-reducing or HIV risk-increasing behaviors. Self-perceived risk is widely considered essential to HIV risk prevention. The Health Belief Model [7] and others include risk perception as an important predictor of risk behaviors. The Health Belief Model includes both disease perception (susceptibility and severity), and behavioral perception (benefit of the action, self-efficacy, cost and barriers to the behavior) as the key determinants in the adoption of healthy habits. For example, the perceived severity and perceived risk have been associated with COVID-19 vaccination uptake in previous studies [8]. Moreover, young people exhibit compulsive, unplanned behaviors that are part of experimentation and identity-building, and often respond to peer pressure [9]. YMSM have sex at younger ages than previous generations and are therefore at greater risk of contracting HIV than their heterosexual counterparts or older MSM [10]. The evidence points to factors related to risky sexual behavior: violence, homophobia (external and internalized), difficulties accepting one’s sexual identity, consumption of alcohol and drugs, relationship dynamics with partners, mental health, and recurrent HIV testing [10,11]. However, in clinical practice, HIV risk is focused on condom use, frequency of unprotected anal sex, sex with an HIV-positive person, sexually transmitted infection (STI) diagnosis, and the use of drugs, without taking into account the psychosocial aspects of sexual health [12,13]. HIV risk perception is crucial to understanding risk behavior [14]; many YMSM are unaware of HIV risks or how to protect themselves [10]. This perception has often been measured and operationalized as a cognitive evaluation of risk, as a probability or a question of the chances of contracting HIV [14]. Various studies have shown that MSM perceived themselves at low risk of HIV infection while they were actually at high risk [13,15]. Furthermore, young people’s perceived low-risk results in decreased condom use and HIV testing, thus increasing their risk of contracting HIV [16].

The epidemic remains poorly defined among YMSM aged 15–24. Little data are available on estimated population size, HIV rates, risk, and protection factors [17]. While a great deal has been written about risk factors and high-risk sex, and despite the widely acknowledged importance of HIV risk perception, very little research has been conducted examining the factors that modulate the underestimation of risk among YMSM. This is a significant gap because it interferes with the specific needs of YMSM in behavioral and biomedical HIV-prevention interventions, such as HIV testing. To our knowledge, there is no research on the perceived HIV risk of Argentinian YMSM. In light of the aforementioned, we aimed to study the conditioning factors of underestimated risk, that is, the discordance between self-perceived and actual HIV risk in YMSM, or a perception of risk lower than the actual risk of HIV infection.

## 2. Materials and Methods

An online, cross-sectional study was conducted using the survey tool Survey Monkey. The study included a convenience sample. The inclusion criteria were YMSM aged 16–24 (in Argentina, they are legally old enough to make decisions by themselves, such as to complete a survey, at 16 years old) without an HIV diagnosis or knowledge of their serostatus. The participant recruitment was carried out on the Facebook and Twitter profiles of Fundación Huésped, an NGO engaged in HIV/AIDS response. A post was shared on these sites inviting users to respond to the online questionnaire. Requests were also sent to LGBT NGOs to disseminate the opportunity to participate. At the same time, an e-mail was sent to young people who had had a negative HIV rapid test at Fundación Huésped, and who had agreed to be contacted for future research.

The questionnaire consisted of 34 closed-ended questions, and the average response time was 20 min. The questions, developed by the research team, were reviewed and crafted in collaboration with young people from Fundación Huésped. The questionnaire was available throughout February 2017. The variables were:

- Outcome: Risk discordance (RD) was defined as the expression of underestimated risk or, rather, a risk perception lower than the actual risk. This variable was dichotomous. Risk discordance was established when the perceived HIV risk—categorized as low, medium, or high—was lower than the actual HIV infection risk—categorized as no risk, low risk, or high risk.
- Actual HIV infection risk (HIV Incidence Risk Index [HIRI]): A proxy for general actual risk, Validated scale specific to MSM with 84% of sensitivity and 45% specificity of HIRI at the 10 cutoff high sensitivity. This index includes seven variables: age, number of male sexual partners, number of times engaged in condomless receptive anal sex, number of HIV-positive partners, number of times engaged in condomless insertive anal sex with an HIV-positive partner, use of methamphetamines, and use of poppers. All the sexual and drug-use questions are related to the previous six months. The scores higher than 10 indicate a significant risk of HIV infection [18]. We classified actual risk as no risk (score 0), low (scores up to 9), and high risk (score of 10 or more). However, as seen in Table 1, the actual index was dichotomized into no/low risk vs. high risk.
- Perceived HIV risk (Perceived HIV Risk Scale [PHRS]): The PRHS is an eight-item sexual behavior scale in English, with the total score ranging from 10 to 40, where 10 is the lowest possible risk perception [14]. It covers various dimensions of risk perception such as cognitive assessment (probability of risk), intuitive judgments, and perceived importance and probability of the risk. The items are presented on a Likert scale, some with four points and others with six. The authors of the scale granted us permission to use it. The scale was translated into Spanish and adapted before use [19]. The perceived HIV risk was constructed as low, medium, and high.
- Internalized homophobia and belonging to the gay community: Adaptation of questions from a previous study [20] to “Sometimes I dislike myself for being gay or bisexual”, “Sometimes I feel guilty for having sex with other men”, “I feel stressed when I have sex with other men”, “Being gay or bisexual makes me feel like part of the gay community”. We used a 3-point scale (1 = Agree, 3 = Disagree).
- HIV testing: Testing history (never, more than one year ago, less than one year ago) and HIV status (negative vs. unknown).
- Sexual behavior: The questions were adapted from previous studies [11,21] related to their sexual network (number of sexual partners in the last 6 months), the use of condoms depending on the partner status (always, almost always, almost never, never), previous STI diagnosis (yes/no), the drugs used to socialize and enjoy sexual practices more (yes/no).
- Relationship status: About the last sexual intercourse, the relationship duration (i.e., less than 3 months, more than 2 years), the venues to meet sexual partners (i.e., online or offline).
- Sexual violence: A history of forced sex was elicited through three questions, i.e., has a man ever forced you to have sex with him? (yes/no).
- Sociodemographic characteristics: Such as age (measured in years and dichotomized into <20 (adolescents) and 20–24 (young adults)), sexual identity (gay, heterosexual, bisexual, MSM, other), educational level (primary, secondary, university, technical studies, finished or unfinished), employment status (employer, self-employed, employee, unpaid family job, student, informal work, no work).

The study and questionnaire were approved by the Research Ethics Committee of Fundación Huésped (Study FH-25). Consent was stated at the beginning of the survey, and it was implicit in accepting to complete the survey. The respondents were allowed to skip any questions that they wished.

The demographic and relevant sample information was described as appropriate (relative frequency for the categorical variables, the mean and standard deviation for the continuous variables). The association between RD and the explanatory variables was analyzed through multivariate logistic regression models. We chose different models to assess the impact on RD of the various aspects (clinical, behavioral, and social) by including the corresponding explanatory variables in the models. A table with the univariable adjustments is available in Table S1. The severity of potential collinearity issues was measured by means of the variance inflation factor (VIF) and no relevant issues were detected. The relationship between HIRI and PHRS scores was studied using correlation analysis. The level of significance was set at bilateral 5%, and all the analyses were carried out using R software (version 3.4.3) using the Available Data Only (ADO) approach (no missing data were imputed) [22]. A table with the percentage of missing data for each variable can be found in Table S2.

## 3. Results

### 3.1. Participant Characteristics and Sexual Health and Behaviors

A total of 405 YMSM with a mean age of 21.08 (SD 2.06) responded to the survey. The participants had a mean of 5.36 (SD 4.98) partners in the last six months, with a relationship duration of less than three months in more than half of the cases. The most frequent relationship was “friend with benefits”. Nearly one-third of the participants reported not knowing their HIV status or not having been tested. Forty percent had been diagnosed with an STI in the past. Two-thirds of the respondents use condoms *always or almost always* in penetrative sex with steady partners. Alcohol and marijuana were most commonly used to socialize and increase the pleasure in sexual encounters. Social networks were used by the majority, with Grindr (42.3%) and Facebook (39.1%) as the most frequent apps for contacting sexual partners. Disco bars (35.3%) and gay bars (30.6%) were the most frequent meeting places. Table 1 shows the demographic and behavioral characteristics of the participants according to the absence or presence of RD.

### 3.2. Discordance of Self-Perceived Risk and Actual HIV Risk, and Associated Factors

Based on the HIRI, a high HIV risk was detected in 251 (62%) of the YMSM, with a mean score of 23.7, while a high self-perceived risk on the PHRS scale was detected in 106 (26.2%) of them. A total of 230 (56.8%) respondents were identified as showing RD. Table 2 describes the habits and sexual health of the YMSM based on the presence or absence of RD, and Table 3 describes the characteristics of the partner relationships and meeting places, based on the absence or presence of RD.

The YMSM who reported college education (aOR = 2.4 [CI 95:1.1–5.5]), consistent condom use with casual partners (aOR = 3.8 [IC 95:1.5–11.0]), use of Growler to meet partners (aOR = 10.38 [IC 95:161–121.94]), frequenting gay bars (aOR = 1.9 [95% CI:1.1–3.5]), and using LSD (aOR = 5.44 [IC 95:1.32–30.29]) showed a higher probability of particularly intense RD. In addition, having sex with a steady partner for more than three months, or with a friend with benefits, increased the odds of RD by two and three times, respectively. Conversely, having a history of syphilis or hepatitis B, an open relationship with a steady partner for more than two years, when the last relationship was with a friend with benefits, and using saunas or bars reduced the likelihood of RD (Table 4).

## 4. Discussion

This is one of the few studies to focus on YMSM—a group that is particularly vulnerable to HIV. This study aimed to explore the self-perception of risk of this population, and compare it with their actual risk of HIV, to understand their underestimation of HIV risk infection. Given that the evidence on this topic has widely studied MSM but not specifically YMSM or Latin American YMSM, we compare our results with the existing literature. Most of the epidemiological studies on MSM do not disaggregate the data by age. Therefore, it is difficult to estimate the population size of YMSM, and this age group may be underrepresented in studies due to the disparities between YMSM and older MSM [4].

More than half of the sample presented risk discordance, or rather, an underestimation of the risk of HIV infection. More than half of the YMSM in this study had a high actual risk, while only a quarter had a high self-perceived risk of HIV infection. A recent secondary analysis conducted in 2018 in Brazil, Mexico, and Peru found similar results: 53.3% of MSM had a high risk of contracting HIV based on their HIRI score, but only 24.7% perceived themselves to be at moderate risk [23]. The self-perceived risk of HIV tends to be lower than the HIRI score [23,24]. Most of the MSM who met ≥10 cutoff criteria of the HIRI score did not perceive themselves to be at a moderate to high HIV risk, but higher HIRI scores were associated with higher self-perceived HIV risk. The HIRI was developed and validated with two cohort studies of adult gay men from the USA in the late 1990s and early 2000s, when the HIV risk factors were different [24]. As a result, the age attributed to the HIRI scores was from the context of adults. Further research is needed to assess the operating characteristics of HIRI scores —mainly age— in predicting HIV in young people in today’s context.

A previous study shows that people with positive HIV or syphilis tests had higher PHRS scores than those who had negative tests [14]. It should be noted that the YMSM in our study had higher self-perceived risk than those who had a negative test in the Napper study, but nearly a third had not been tested for HIV. In fact, our results suggest that having or not having had a recent HIV test was not associated with HIV-risk discordance or underestimation of being at risk for HIV infection, despite the HIV test being a key tool in identifying people at risk of HIV infection and connecting them to care [25]. Young Latinos in the USA had the lowest rates of HIV testing of all ethnic groups [26]. According to the literature, underestimated self-risk is related to actual HIV infection risk, and only those who feel at risk go for testing [25,27]. However, it is likely that YMSM consider HIV testing to be screening rather than a preventive behavior, so HIV testing is self-initiated when they acknowledge their HIV vulnerability. In 2018, a cross-sectional study found that 70% (n = 157) of Argentinian YMSM reported no prior HIV test [28]. Self-perception of being at risk and HIV testing depends on several factors, including age. A Brazilian study shows that YMSM were more likely to have condomless receptive anal sex and transactional sex but had a lower perceived HIV risk and less HIV testing than older MSM [29].

Some psychosocial predictors of an increased RD, or underestimation of HIV risk infection, include not experiencing internalized homophobia, certain partner statuses, using LSD, using Growler, and visiting gay bars to meet sexual partners. On the other hand, there are other psychosocial aspects that predict a lower RD, such as having had an STI diagnosis, other partner statuses, and other meeting places, among others.

LSD use also increases the underestimation of HIV risk infection, although, in other studies, the association has been with drug use in general [4,20]. Drug use and poly-drug use are common among YMSM living in Buenos Aires [28]. The most frequently used drugs were marijuana and cocaine. There is evidence that adolescent drug use is a direct predictor of risky sexual behavior [4,20], and it is also associated with a low self-perceived risk of HIV infection [30].

More than 70% of YMSM in our study do not present traits of internalized homophobia, a growing trend among young people [31]. This was a factor in underestimating their risk of HIV infection. In the case of internalized homophobia, internalized social guilt and the sociocultural organization of gender relations play an essential role in shaping risk perception marked by homophobic conditions [32]. It reinforces a type of practice—an increased number of sexual partners, unprotected insertive sex [31]—that is more in line with socially accepted masculinity [33], following heteronormative stereotypes [34]. Evidence shows that internalized homophobia in YMSM was associated with more partners and risky sexual practices, particularly insertive sex [31]. In contrast, the evidence among adult MSM is controversial [35,36,37]. It would seem reasonable to think that YMSM without internalized homophobia would opt for safer practices, but our data suggest that those with RD engage in more risky practices because they have a high actual risk. This discordance or underestimation of HIV risk should be studied in depth. Future research should assess whether or not the presence of traits of internalized homophobia intervene in the relationship between an underestimation of HIV risk, self-perceived HIV risk, and actual risk in YMSM.

In this study, we found that a prior diagnosis of an STI decreases RD, likely because it increases the perception of vulnerability. Although YMSM aged 20–24 years have high rates of STIs [6], less than half of them report having undergone STI testing in the past year [38].

Having concurrent sexual partners is common among YMSM [37], and it is more common among YMSM to have unprotected anal sex with casual partners while in a steady sexual relationship [10,39]. The partner status plays several roles in YMSM RD. The results of this study suggest that maintaining an open relationship with a steady partner for more than two years or having last had sex with a friend with benefits reduces discordance. In contrast, having a steady partner or a friend with benefits for more than three months increases discordance. There is a social belief that casual relationships are the ones causing HIV transmission [40] even though there is no evidence to support it. The evidence suggests that most new HIV infections in YMSM occur in the context of steady relationships because they have more condomless sex in committed or closer relationships than with casual partners [40]. Something similar happens with adult MSM because condom use within committed relationships is variable given that HIV risk perception is low [41].

Considering the relationship to be “serious” was associated with an almost eightfold increase in the rate of condomless anal intercourse [35] among YMSM. It remains unclear why YMSM had more condomless sex with their main partners compared to adult MSM but classifying a relationship as steady was the most significant predictor of having condomless anal intercourse among YMSM [36]. A relationship is classified as stable or “safe” when the partner is trusted, and one feels that their partner is not dangerous or when one knows their partner personally [39]. Because one knows their friend and steady partner personally and trusts them, they do not perceive themselves as at risk. It also remains unclear how the relationship status changes over time and how a relationship becomes closer and is eventually perceived as “safer”. A longitudinal study found that YMSM classified a relationship as serious after less than six months of sexual contact, while our participants classified a relationship as serious after less than three months of sexual contact [40]. Considering this evidence, the perceived risk of HIV of Argentinian YMSM places them in real danger of contracting HIV. Thus, biomedical HIV preventive strategies, such as PrEP or behavioral interventions, should be performed to deconstruct the social belief that sex with steady partners or friends is safer than with casual partners.

Frequenting gay bars increased the RD. The association between going to gay bars and high-risk sex has been studied in the past. Frequenting such bars contributes to being part of the community, even though they tend to be more permissive of drug use and specific sexual behaviors (such as underground private sex parties or barebacking subculture) [39]. HIV-risk behaviors are normalized, thus reducing the self-perception of HIV risk. On the other hand, evidence suggests that YMSM participate differently in the gay communities [39]. The widespread use of the Internet among MSM has led to a decline in gay infrastructure, visibility, and community identification across gay communities [42]. The internet has taken on the role of the traditional “gay bar”, providing a popular way for MSM to socialize and meet sexual partners [43]. YMSM use many apps and different settings to meet people, but there was only increased discordance among those using Growler (n = 16), in which case the risk perception did not correspond with the actual risk. Evidence demonstrates that MSM who seek out partners online have more sex and more partners, but this was not associated with greater risk because there is also greater distrust and suspicion of partners met online [44]. The number of sexual partners increases the perception of HIV risk and is also a predictor of risky sexual behavior in this group [40,45].

The main limitations of this study are the data collection method, the use of convenience sampling, and the use of scales not validated in Spanish or YMSM. We chose to use an online questionnaire disseminated via the social network profiles of Fundación Huésped to provide easier access for the study population by allowing them to respond on any device. This may be a limitation when generalizing the data because we do not know how many people it reached or the response rate. The questionnaire was lengthy. It took about 20 min to complete but included validated tools, unlike other studies of this kind. Another limitation was related to the HIRI, which was validated in Spanish but not in the population of Argentinian MSM. Age has been a significant factor when calculating the actual risk in this study population. On the HIRI, eight points are allocated for ages ranging from 18 to 28 years. Thus, very little more is needed for young people to be at high risk. It is unknown what score should be used for targeting high-risk YMSM, so more research is needed to fill this gap.

## 5. Conclusions

The discordance between self-perceived and actual HIV risk increases the vulnerability of YMSM to HIV, which should be corrected by addressing the increasing predictive factors. The underestimation of HIV risk is mainly associated with psychosocial aspects such as not experiencing internalized homophobia, having a steady partner or a friend with benefits for over three months, the use of Growler or specific venues to meet sexual partners, and LSD use. These aspects should be included in HIV risk-reduction strategies focusing on psychosocial factors. It is important that future research characterizes the complexity of relationships between YMSM, identifying the processes by which relationship status changes over time and becomes closer, as well as its link to underestimating HIV risk.

Our study supports the underestimation of HIV risk in almost 50% of YMSM in Argentina. Therefore, culturally tailored interventions should be conducted for YMSM at both the structural and individual levels. At the structural level, young men who are particularly vulnerable to HIV infection should be involved in designing strategies that (a) use new technologies to provide information about the underestimation of HIV risk in YMSM; (b) facilitate access to PrEP; (c) facilitate access to HIV screening and counseling; (d) involve primary care provider in screening young patients for HIV testing based on their specific risk behaviors, rather than their self-perception of risk. The novel contribution of the results of this study reframes the sensitivity of the instruments used to measure the risk of HIV in YMSM, which are the basis for guiding prevention strategies. It seems that, in YMSM, the psychosocial aspects have an important weight in estimating said risk.

At the individual level, HIV prevention messages aimed at YMSM should be addressed to promote acknowledgment of one’s vulnerability and encourage health-seeking behaviors. Health professionals should reconsider adapting the instruments to measure the risk of HIV in YMSM and be aware of the psychosocial aspects to succeed in communicating HIV prevention messages.

## Figures and Tables

**Table 1:** Sociodemographic characteristics, internalized homophobia, HIV risk perception (PHRS) and HIV risk (HIRI) based on the absence or presence of HIV risk discordance. *p*-values from Student’s t-test for continuous variables and chi-squared test for categorical variables.

Demographic and Clinical Variables	All Participants n = 405 Frequency (%)	Risk Concordance n = 175 Frequency (%)	Risk Discordance n = 230 Frequency (%)	*p*-Value
**Mean age (SD)**	21.08 (2.06)	21.9 (2.1)	21.8 (2.1)	0.547
<20 years	110 (27.2)	46 (26.3)	64 (27.8)	0.816
20–24 years	295 (72.8)	129 (73.7)	166 (72.2)	
**Sex**				0.637
Men	401 (99.0)	174 (99.4)	227 (98.7)	
Transgender Men	4 (1.0)	1 (0.6)	3 (1.3)	
**Place of residence (n = 372)**				0.931
City (Buenos Aires)	96 (23.7)	42 (24.0)	54 (23.5)	
Suburban city	81 (20.0)	32 (18.3)	49 (21.3)	
Province (Buenos Aires)	57 (14.1)	25 (14.3)	32 (13.9)	
Other provinces	138 (34.1)	60 (34.3)	78 (33.9)	
Unknown	33 (8.1)	16 (9.1)	17 (7,4)	
**Educational level (n = 402)**				0.112
Primary studies finished	2 (0.5)	1 (0.6)	1 (0.4)	
Secondary studies unfinished	19 (4.7)	11 (6.3)	8 (3.5)	
Secondary studies finished	36 (8.9)	9 (5.1)	27 (11.7)	
University degree/Trade school unfinished	250 (61.7)	110 (62.9)	140 (60.9)	
University degree/Trade school finished	95 (23.5)	42 (24.0)	53 (23.0)	
Unknown	3 (0.7)	2 (1.1)	1 (0.4)	
**Employment status (n = 402)**				0.654
Employer	10 (2.5)	4 (2.3)	6 (2.6)	
Self-employed	24 (5.9)	7 (4.1)	17 (7.4)	
Employee	137 (33.8)	63 (36.0)	74 (32.2)	
Unpaid family work	1 (0.2)	1 (0.6)	0 (0.0)	
Student	192 (47.4)	79 (45.1)	113 (49.1)	
Informal work	17 (4.2)	8 (4.6)	9 (3.9)	
No work	21 (5.2)	10 (5.7)	11 (4.8)	
Unknown	3 (0.7)	3 (1.7)	0	
**Living (n = 396)**				0.654
With parents	234 (57.8)	103 (58.9)	131 (57.0)	
Alone in an apartment	91 (22.5)	39 (22.3)	52 (22.6)	
Alone in residence	4 (1.0)	3 (1.7)	1 (0.4)	
With friends	23 (5.7)	7 (4.0)	16 (7.0)	
With partner	26 (6.4)	10 (5.7)	16 (7.0)	
Other	18 (4.4)	7 (4.0)	11 (4.8)	
Unknown	9 (2.2)	6 (3.4)	3 (1.3)	
**Sexual relations with (n = 400)**				0.827
Men	357 (88.1)	157 (89.7)	200 (87.0)	
Women	2 (0.5)	1 (0.6)	1 (0.4)	
Men & women	30 (7.4)	10 (5.7)	20 (8.7)	
Transgender women	3 (0.7)	1 (0.6)	2 (0.9)	
Men & women & transgender women	8 (2.0)	3 (1.7)	5 (2.2)	
Unknown	5 (1.2)	3 (1.7)	2 (0.9)	
**Sexual Identity (n = 404)**				0.440
Gay	307 (75.8)	132 (75.4)	175 (76.1)	
Bisexual	40 (9.9)	20 (11.4)	20 (8.7)	
Heterosexual	11 (2.7)	2 (1.1)	9 (3.9)	
MSM	42 (10.4)	19 (10.9)	23 (10.0)	
Other	4 (1.0)	2 (1.1)	2 (0.9)	
Unknown	1 (0.2)	0	1 (0.4)	
Internalized Homophobia				
*Sometimes I dislike myself for being gay or bisexual*				0.030
Agree	81 (20.0)	43 (24.6)	38 (16.5)	
Indifferent	43 (10.6)	23 (13.1)	20 (8.7)	
Disagree	279 (68.9)	109 (62.3)	170 (73.9)	
Unknown	2 (0.5)	0	2 (0.9)	
*Sometimes I feel guilty for having sex with other men*				0.022
Agree	80 (19.8)	44 (25.1)	36 (15.7)	
Indifferent	39 (9.6)	20 (11.4)	19 (8.3)	
Disagree	285 (70.4)	111 (63.4)	174 (75.7)	
Unknown	1 (0.2)	0	1 (0.4)	
*I feel stressed when I have sex with other men*				0.124
Agree	67 (16.5)	34 (19.4)	33 (14.3)	
Indifferent	59 (14.6)	30 (17.1)	29 (12.6)	
Disagree	278 (68.6)	111 (63.4)	167 (72.6)	
Unknown	1 (0.2)	0	1 (0.4)	
**Belonging to gay community**				
*Being gay or bisexual makes me feel like part of the gay community*				0.638
Agree	127 (31.4)	52 (29.7)	75 (32.6)	
Indifferent	177 (43.7)	75 (42.9)	102 (44.3)	
Disagree	100 (24.7)	47 (26.9)	53 (23.0)	
Unknown	1 (0.2)	1 (0.6)	0	
**HIV Risk Perception Scale (PHRS)**				<0.001
[10,22] Low risk	138 (34.1)	1 (0.6)	137 (59.6)	
[22,28] Medium risk	161 (39.7)	68 (38.9)	93 (40.4)	
[28,39] High risk	106 (26.2)	106 (60.6)	0 (0)	
**HIV Incidence Risk Index (HIRI)**				
[1,9] Low risk/[0] No risk	154 (38.0)	-	-	
[>10] High Risk	251 (62.0)	-	-	

**Table 2:** Sexual behavior and sexual health based on the absence or presence of HIV risk discordance. *p*-values from Student’s t-test for continuous variables and chi-squared test for categorical variables.

Sexual Behavior and Sexual Health Variables	All Participants n = 405 Frequency (%)	Risk Concordance n = 175 Frequency (%)	Risk Discordance n = 230 Frequency (%)	*p*-Value
**Sexual Network**				
Number of sexual partners in the last 6 months mean (±SD *)	5.36 (±4.98)	5.18 (±4.0)	5.50 (±5.6)	0.506
Frequency of **condom use and partner status**				
With steady partner				0.671
Always/Almost always	255 (63.0)	113 (64.6)	142(61.7)	
Almost never/Never	150 (37.0)	62 (35.4)	88 (38.2)	
With casual partners				0.031
Always/Almost always	341 (84.2)	139 (79.4)	202 (87.8)	
Almost never/Never	64 (15.8)	36 (20.6)	28 (12.2)	
With friends with benefits				0.612
Always/Almost always	340(84.0)	144 (82.3)	196 (85.2)	
Almost never/Never	65 (16.0)	31 (17.7)	34 (14.8)	
Oral sex (fellatio and black kiss)	56 (13.8)	27 (15.4)	29 (12.6)	0.503
**HIV test**				0.223
Never	127 (31.4)	58 (33.1)	69 (30.0)	
More than one year ago	108 (26.7)	39 (22.3)	69 (30.0)	
Less than one year ago	168 (41.5)	77 (44.0)	91 (39.6)	
Unknown	2 (0.5)	1 (0.6)	1 (0.4)	
**HIV status**				0.684
Negative	274 (67.6)	116 (66.3)	158 (68.7)	
Unknown	131 (32.4)	59 (33.7)	72 (31.3)	
**Past STI diagnosis**				
Syphilis	30 (7.6)	18 (10.7)	12 (5.3)	0.071
Gonorrhea	27 (6.8)	17 (9.9)	10 (4.4)	0.046
Genital warts	79 (20.0)	41 (24.4)	38 (16.7)	0.079
Hepatitis A	14 (3.6)	8 (4.8)	6 (2.7)	0.400
Hepatitis B	11(2.8)	8 (4.8)	3 (1.3)	0.061
Hepatitis C	2 (0.5)	0	2	0.510
**Violence**				
*Have you ever had sex in exchange for money, help, lodging, protection or gifts?*	54 (13.3)	26 (14.9)	28 (12.2)	0.541
*Have you ever offered sex in exchange for money, help, lodging, protection or gifts?*	23 (5.7)	11 (6.3)	12 (5.2)	0.824
*Has a man ever forced you to have sex with him?*	32 (7.9)	14 (8.0)	18 (7.8)	1.000
**Participation in chemsex parties**	19 (4.7)	8 (4.6)	11 (4.8)	1.000
**Sex with HIV-positive men**				0.011
Always/Almost always	19 (4.7)	14 (8.1)	5 (2.2)	
Almost never/Never	382 (95.3)	158 (91.9)	224 (97.8)	
**Use of drugs to socialize and enjoys sexual practices more**				
Alcohol	280 (69.5)	113 (64.9)	167 (72.9)	0.106
Marijuana	162 (40.3)	71 (40.8)	91 (39.9)	0.938
Poppers	32 (8.0)	14 (8.1)	18 (8.0)	1.000
Cocaine	20 (5.0)	11 (6.4)	9 (4.0)	0.377
Ecstasy, MDMA or amphetamines	24 (6.0)	11 (6.4)	13 (5.8)	0.968
LSD	29 (7.3)	10 (5.8)	19 (8.4)	0.436
Viagra, Cialis	18 (4.5)	8 (4.7)	10 (4.4)	1.000
GHB/GBL (liquid ecstasy)	11 (2.8)	3 (1.7)	8 (3.5)	0.363

* SD: Standard Deviation.

**Table 3:** Characteristics of relationships and meeting places based on the absence or presence of HIV risk discordance. *p*-values from Student’s t-test for continuous variables and chi-squared test for categorical variables.

Relationships Status and Meeting Place Variables	All Participants n = 405 Frequency (%)	Risk Concordance n = 175 Frequency (%)	Risk Discordance n = 230 Frequency (%)	*p*-Values
**Most common type of sexual relationship**				0.004
Closed steady partner	101 (24.9)	33 (19.5)	68 (30.2)	
Open steady partner	30 (7.4)	7 (4.1)	23 (10.2)	
Occasional partners	159 (39.3)	80 (47.3)	79 (35.1)	
Friends with benefits	104 (25.7)	49 (29.0)	55 (24.4)	
Unknown	11 (2.7)			
**If you have a closed steady partner, this relationship lasted**				0.002
Less than 3 months	217 (53.6)	112 (64.0)	105 (45.7)	
Between 3 months and 1 year	85 (21.0)	26 (14.9)	59 (25.7)	
Between 1 and 2 years	51 (12.6)	16 (9.1)	35 (15.2)	
More than 2 years	52 (12.8)	21 (12.0)	31 (13.5)	
**If you have friends with benefits, this relationship lasted**				0.095
Less than 3 months	242 (59.8)	116 (66.3)	126 (54.8)	
Between 3 months and 1 year	78 (19.3)	31 (17.7)	47 (20.4)	
Between 1 and 2 years	37 (9.14)	13 (7.4)	24 (10.4)	
More than 2 years	48 (11.9)	15 (8.6)	33 (14.3)	
**Last sexual relationship was**				0.153
Open steady partner	115 (28.7)	42 (24.3)	73 (32.0)	
Closed steady partner	24 (6.0)	8 (4.6)	16 (7.0)	
Friends with benefits	172 (42.9)	84 (48.6)	88 (38.6)	
Occasional partners	90 (22.4)	39 (22.5)	51 (22.4)	
**Venues to meet sexual partners (social media)**				
Badoo	40 (10.1)	15 (8.8)	25 (11.1)	0.560
Grindr	170 (42.3)	79 (45.7)	91 (39.7)	0.276
Manhunt	54 (13.3)	29 (16.6)	25 (10.9)	0.127
Growler	16 (4.0)	4 (2.3)	12 (5.2)	0.222
Contactossex	9 (2.3)	6 (3.5)	3 (1.3)	0.180
Tinder	107 (26.5)	48 (27.6)	59 (25.7)	0.747
Facebook	156 (39.1)	72 (41.6)	84 (37.2)	0.424
Gay chats	43 (10.6)	21 (12.0)	22 (9.6)	0.542
**Meeting places**				
Gay bar	123 (30.6)	46 (26.4)	77 (33.8)	0.141
Bar	100 (24.7)	47 (26.9)	53 (23.0)	0.444
Sex shops	7 (1.7)	2 (1.2)	5 (2.2)	0.704
Disco bar	143 (35.3)	61 (34.9)	82 (35.7)	0.951
Sauna	20 (5.0)	12 (6.9)	8 (3.5)	0.189
Cruising	21 (5.2)	7 (4.0)	14 (6.1)	0.464
Public toilets	29 (7.2)	12 (6.9)	17 (7.4)	0.990
Sex parties	15 (3.7)	6 (3.5)	9 (4.0)	1.000

**Table 4:** Multivariate logistic regression of factors significantly associated with HIV risk discordance.

Variables	aOR * (CI 95%)	*p* Value
Internalized Homophobia		
*Sometimes I dislike myself for being gay or bisexual* (Disagree vs. Agree)	1.8 (1.1–2.9)	0.02
*I feel stressed when I have sex with other men* (Disagree vs. Agree)	1.9 (1.2–3.12)	0.01
**Sexual Health**		
Syphilis (Yes vs. No)	0.40 (0.2–0.9)	0.036
Hepatitis B (Yes vs. No)	0.1 (0.01–0.7)	0.042
**Condom use**		
Condom use during penetration with casual partners (Always vs. Almost never)	3.8 (1.5–11.0)	0.003
**Drug use**		
LSD (Consume vs. Not consume)	5.4 (1.3–30.3)	0.03
**Relationship**		
If you have a closed steady partner, this relationship lasted (From 3 months to 1-year vs. Less than 3 months)	2.2 (1.1–4.3)	0.024
If you have an open steady partner, this relationship lasted (More than 2 years vs. Less than 3 months)	0.3 (0.1–0.8)	0.02
If you have friends with benefits, this relationship lasted (More than 2 years vs. Less than 3 months)	3.1 (1.4–7.2)	0.005
Last sexual relationship was (Friend with benefits vs. Closed steady partner)	0.6 (0.4–0.97)	0.04
**Strategy for meeting sexual partners**		
Growler (Yes vs. No)	10.4 (1.6–121.9)	0.030
Gay bar (Yes vs. No)	1.9 (1.1–3.5)	0.034
Bar (Yes vs. No)	0.5 (0.3–0.98)	0.040
Saunas (Yes vs. No)	0.1 (0.0–0.4)	0.004

* aOR: Adjusted odds ratio.

## Data Availability

The data that support the findings of this study are available upon request from the corresponding author. The data are not publicly available due to privacy or ethical restrictions.

## Supplementary Materials

The following supporting information can be downloaded at: https://www.mdpi.com/article/10.3390/ijerph192215269/s1. Table S1. Univariable model of factors significantly associated with HIV risk discordance; Table S2. Number and percentatge of missing data for each variable.

## References

[1] Dirección de Respuesta al VIH ITS, Hepatitis Virales y Tuberculosis (2020). Boletín N° 37. Respuesta al VIH y Las ITS En La Argentina. https://bancos.salud.gob.ar/sites/default/files/2020-11/Boletin%20VIH%202020%20final%20V2.pdf.

[2] Dirección de Sida y ETS. Ministerio de la Salud. Presidencia de la Nación (2017). Boletín Sobre El VIH, Sida e ITS en La Argentina. https://bancos.salud.gob.ar/recurso/boletin-sobre-el-vih-sida-e-its-en-la-argentina-ndeg-34.

[3] UNAIDS (2022). IN DANGER: UNAIDS Global AIDS Update.

[4] Coelho L.E., Torres T.S., Veloso V.G., Grinsztejn B., Jalil E.M., Wilson E.C., McFarland W. (2021). The Prevalence of HIV Among Men Who Have Sex With Men (MSM) and Young MSM in Latin America and the Caribbean: A Systematic Review. AIDS Behav..

[5] Centers for Disease Control and Prevention (2021). Dear Colleague. What’s New. https://www.cdc.gov/hiv/policies/dear-colleague/dcl/052721.html.

[6] Centers for Disease Control and Prevention (2021). HIV and Youth. Last Updated 2 July 2021. https://www.cdc.gov/hiv/group/age/youth/.

[7] Champion V.L., Skinner C.S. (2008). The health belief model. Health Behav. Health Educ. Theory Res. Pract..

[8] Wong M.C.S., Wong E.L.Y., Huang J., Cheung A.W.L., Law K., Chong M.K.C., Ng R.W.Y., Lai C.K.C., Boon S., Lau J.T.F. (2021). Acceptance of the COVID-19 vaccine based on the health belief model: A population-based survey in Hong Kong. Vaccine.

[9] Allan-Blitz L.T., Mena L.A., Mayer K.H. (2021). The ongoing HIV epidemic in American youth: Challenges and opportunities. mHealth.

[10] World Health Organization (2015). HIV and Young Men Who Have Sex with Men. Technical Brief. https://www.who.int/publications/i/item/WHO-HIV-2015.8.

[11] Fernández-Dávila P., Zaragoza Lorca K. (2011). Hombres jóvenes que tienen sexo con hombres: ¿Un colectivo en alto riesgo para la infección por el VIH?. Gac. Sanit..

[12] Kabapy A.F., Shatat H.Z., Abd El-Wahab E.W. (2020). Attributes of HIV infection over decades (1982–2018): A systematic review and meta-analysis. Transbound. Emerg. Dis..

[13] Freeborn K., Portillo C., Boyer C.B., Santos G.M. (2020). Misclassification of sexual health risks in a self-identified low risk cohort of men who have sex with men (MSM) enrolled in a community based PrEP program. AIDS Care.

[14] Napper L.E., Fisher D.G., Reynolds G.L. (2012). Development of the perceived risk of HIV scale. AIDS Behav..

[15] Kesler M.A., Kaul R., Liu J., Loutfy M., Gesink D., Myers T., Remis R.S. (2016). Actual sexual risk and perceived risk of HIV acquisition among HIV-negative men who have sex with men in Toronto, Canada. BMC Public Health.

[16] Li Y.-H., Mgbere O., Abughosh S., Chen H., Cuccaro P., Smesny A., Essien E.J. (2019). Assessment of sexually transmitted disease/HIV risk among young African Americans: Comparison of self-perceived and epidemiological risks utilizing ecodevelopmental theory. HIV/AIDS.

[17] Mwaniki S.W., Mugo P.M., Palanee-Phillips T. (2021). Project BESPOKE (Integrated Bio-Behavioral Assessment of HIV and STI Among Young Tertiary Student Men Who Have Sex With Men in Nairobi, Kenya): A Respondent-Driven Sampling Survey Protocol. Front. Public Health.

[18] Smith D.K., Pals S.L., Herbst J.H., Shinde S., Carey J.W. (2012). Development of a clinical screening index predictive of incident HIV infection among men who have sex with men in the United States. J. Acquir. Immune. Defic. Syndr..

[19] Sousa V.D., Rojjanasrirat W. (2011). Translation, adaptation and validation of instruments or scales for use in cross-cultural health care research: A clear and user-friendly guideline. J. Eval. Clin. Pract..

[20] Flores S.A., Mansergh G., Marks G., Guzman R., Colfax G. (2009). Gay identity-related factors and sexual risk among men who have sex with men in San Francisco. AIDS Educ. Prev..

[21] Eluwa G.I., Sylvia A., Luchters S., Ahonsi B. (2015). HIV Risk Perception and Risk Behaviors among Men Who Have Sex with Men in Nigeria. J. AIDS Clin. Res..

[22] R Core Team (2018). R: A Language and Environment for Statistical Computing.

[23] Assaf R.D., Konda K.A., Torres T.S., Vega-Ramirez E.H., Elorreaga O.A., Diaz-Sosa D., Diaz S.D., Pimenta C., Robles R., Medina-Mora M.E. (2021). Are men who have sex with men at higher risk for HIV in Latin America more aware of PrEP?. PLoS ONE.

[24] Wilton J., Mishra S., Tan D.H. (2017). Considerations for Using the HIRI-MSM Screening Tool to Identify MSM Who Would Benefit Most From PrEP. JAIDS.

[25] Price J.T., Rosenberg N.E., Vansia D., Phanga T., Bhushan N.L., Maseko B., Brar S.K., Hosseinipour M.C., Tang J.H., Bekker L.-G. (2018). Predictors of HIV, HIV Risk Perception, and HIV Worry Among Adolescent Girls and Young Women in Lilongwe, Malawi. J. Acquir. Immun. Defic. Syndr..

[26] Adebayo O.W., de Santis J.P., Gattamorta K.A., Villegas N.A. (2020). Facilitators of self-initiated HIV testing among youths: A qualitative study. J. Nurs. Res..

[27] Ma M., Malcolm L.R. (2016). Cultural influences on HIV testing among Latino youth. Cult. Health. Sex..

[28] Balán I.C., Frasca T., Pando M.A., Marone R.O., Barreda V., Dolezal C., Carballo-Diéguez A., Ávila M.M. (2018). High Substance Use and HIV Risk Behavior Among Young Argentine Men Who Have Sex with Men. AIDS Behav..

[29] Torres T.S., Luz P.M., DE Boni R.B., De Vasconcellos M.T.L., Hoagland B., Garner A., Moreira R.I., Veloso V.G., Grinsztejn B. (2019). Factors associated with PrEP awareness according to age and willingness to use HIV prevention technologies: The 2017 online survey among MSM in Brazil. AIDS Care.

[30] Evangeli M., Baker L.L.E., Pady K., Jones B., Wroe A.L. (2016). What leads some people to think they are HIV-positive before knowing their diagnosis? A systematic review of psychological and behavioural correlates of HIV-risk perception. AIDS Care.

[31] Meanley S., Egan J.E., Bauermeister J.A. (2018). Policing Heteronormativity and Sexual Risk-Taking Among Young Adult Men Who Have Sex with Men in the Detroit Metro Area. AIDS Behav..

[32] Salazar X., Caceres C., Maiorana A., Rosasco A.M., Kegeles S., Coates T. (2006). Influencia del contexto sociocultural en la percepción del riesgo y la negociación de protección en hombres homosexuales pobres de la costa peruana. Cad. Saude Publica.

[33] Fields E.L., Bogart L.M., Smith K.C., Malebranche D.J., Ellen J., Schuster M.A. (2015). “I always felt I had to prove my manhood”: Homosexuality, masculinity, gender role strain, and HIV risk among young Black men who have sex with men. Am. J. Public Health.

[34] Puckett J.A., Newcomb M.E., Ryan D.T., Swann G., Garofalo R., Mustanski B. (2017). Internalized Homophobia and Perceived Stigma: A Validation Study of Stigma Measures in a Sample of Young Men who Have Sex with Men. Sex. Res. Soc. Policy.

[35] Crepaz N., Marks G., Mansergh G., Murphy S., Miller L., Appleby P.R. (2000). Age-related risk for HIV infection in men who have sex with men: Examination of behavioral, relationship, and serostatus variables. AIDS Educ. Prev..

[36] Newcomb M., Ryan D., Garofalo R., Mustanski B. (2014). The effects of sexual partnership and relationship characteristics on three sexual risk variables in young men who have sex with men. Arch. Sex. Behav..

[37] Mitchell J.W., Petroll A.E. (2013). Factors Associated with Men in HIV-Negative Gay Couples Who Practiced UAI Within and Outside of Their Relationship. AIDS Behav..

[38] Centers for Disease Control and Prevention (2020). HIV Infection Risk, Prevention, and Testing Behaviors among Persons Who Inject Drugs—National HIV Behavioral Surveillance: Injection Drug Use, 23 U.S. Cities, 2018. http://www.cdc.gov/hiv/library/reports/hiv-surveillance.html.

[39] Mustanski B.S., Newcomb M.E., Du Bois S., Garcia S.C., Grov C. (2011). HIV in Young Men Who Have Sex with Men: A Review of Epidemiology, Risk and Protective Factors, and Interventions. J. Sex. Res..

[40] Mustanski B., Newcomb M.E., Clerkin E.M. (2011). Relationship Characteristics and Sexual Risk-Taking in Young Men Who Have Sex with Men. Health Psychol..

[41] Brady S.S., Iantaffi A., Galos D.L., Rosser B.R.S. (2013). Open, Closed, or In Between: Relationship Configuration and Condom Use among Men Who Use the Internet to Seek Sex with Men. AIDS Behav..

[42] Simon Rosser B.R., West W., Weinmeyer R. (2008). Are gay communities dying or just in transition? Results from an international consultation examining possible structural change in gay communities. AIDS Care.

[43] Grosskopf N.A., Harris J.K., Wallace B.C., Nanin J.E. (2011). Online Sex-Seeking Behaviors of Men Who Have Sex with Men in New York City. Am. J. Mens Health.

[44] Macapagal K., Moskowitz D., Li D.H., Carrión A., Bettin E., Fisher C., Mustanski B. (2018). Hookup App Use, Sexual Behavior, and Sexual Health Among Adolescent Men Who Have Sex with Men in the United States. J. Adolesc. Health.

[45] Rosenberg E.S., Sullivan P.S., A DiNenno E., Salazar L.F., Sanchez T.H. (2011). Number of casual male sexual partners and associated factors among men who have sex with men: Results from the National HIV Behavioral Surveillance system. BMC Public Health.

