# Supplementary material for: Assessing the Underestimation of HIV Risk Infection among Young Men Who Have Sex with Men in Argentina
Source: Int J Environ Res Public Health. 2022 Nov 18;19(22):15269. doi: 10.3390/ijerph192215269 (PMC9690491; doi:10.3390/ijerph192215269)
Supplement: Supplementary file 1 [file ijerph-19-15269-s001.zip › ijerph-1980359-supplementary.pdf]

Supplementary material S1. Univariable model of factors significantly associated with HIV risk discordance

Variables	[a]aOR ([b]CI 95%)	P-value
**Internalized homophobia**		
Sometimes I dislike myself for being gay or bisexual (Indifferent *vs* Agree	0.98 (0.47-2.07)	0.966
Sometimes I dislike myself for being gay or bisexual (Disagree *vs* Agree)	1.76 (1.07-2.91)	0.025
I feel guilty when I have sex with other men (Indifferent *vs* Agree)	1.16 (0.54-2.51)	0.703
I feel guilty when I have sex with other men (Disagree *vs* Agree)	1.92 (1.16-3.17)	0.011
**Sexual health**		
Syphilis (Yes *vs* No)	0.47 (0.21-0.99)	0.050
Hepatitis B (Yes *vs* No)	0.27 (0.06-0.95)	0.057
**Condom use**		
Condom use during penetration with casual partners (Always *vs* Almost never)	1.87 (1.09-3.22)	0,023
**Drug use**		
LSD (Consume *vs* No consume)	1.48 (0.68-3.40)	0.333
**Relationship**		
If you have a closed steady partner, this relationship lasted (Between 3 months and 1 year *vs* Less than 3 months)	2.42 (1.43-4.17	0.001
If you have a closed steady partner, this relationship lasted (Between 1 and 2 years *vs* Less than 3 months)	2.33 (1.24-4.56)	0.010
If you have a closed steady partner, this relationship lasted (More than 2 years *vs* Less than 3 months)	1.57 (0.86-2.94)	0.148
If you have an open steady partner, this relationship lasted (Between 3 months to 1 year *vs* Less than 3 months)	1.71 (0.92-3.32)	0.100
If you have an open steady partner, this relationship lasted (Between 1 year to 2 year *vs* Less than 3 months)	1.66 (0.77-3.81	0.210
If you have an open steady partner, this relationship lasted (More than 2 years *vs* Less than 3 months)	0.88 (0.43-1.79)	0.719
If you have friends with benefits, this relationship lasted (Between 3 months and 1 year *vs* Less than 3 months)	1.40 (0.83-2.36)	0.208
If you have friends with benefits, this relationship lasted (Between 1 and 2 years *vs* Less than 3 months)	1.70 (0.84-3.58)	0.149
If you have friends with benefits, this relationship lasted (More than 2 years *vs* Less than 3 months)	2.03 (1.06-4.02)	0.036
Last sexual relationship was (Open steady *vs* Closed steady partner)	1.15 (0.46-3.05)	0.767
Last sexual relationship was (Occasional *vs* Closed steady partner)	0.60 (0.37-0.97)	0.040
Last sexual relationship was (Friend with benefits *vs* Closed steady partner)	0.75 (0.43-1.32)	0.323
**Strategy for meeting sexual partners**		
Growler (Yes *vs* No)	2.33 (0.79-8.44)	0.150
Gay bar (Yes *vs* No)	1.42 (0.92-2.20)	0.115
Bar (Yes *vs* No)	0.82 (0.52-1.29)	0.378
Saunas (Yes *vs* No)	0.49 (0.19-1.22)	0.129

[a] aOR: Adjusted Odds ratio, [b]CI: Confidence Interval

Supplementary Material S2. Number and percentatge of missing data for each variable

Variables	Missing Data (n)	Missing Data. (%)
**Demographic variables**		
Age	0	0%
Sex	0	0%
Place of residence	33	8.15%
Educational level	3	0.74%
Employment status	3	0.74%
Living	9	2.22%
Sexual relations with	5	1.23%
Sexual Identity	1	0.25%
Internalized Homophobia		
Sometimes I dislike myself for being gay or bisexual	2	0.49%
Sometimes I feel guilty for having sex with other	1	0.25%
I feel stressed when I have sex with other men	1	0.25%
Belonging to gay community	1	0.25%
HIV Risk Perception Scale (PHRS)	0	0%
HIV Incidence Risk Index (HIRI])	0	0%
**Sexual behavior and sexual healh**		
Frecuency of condom use and partner status	0	0%
HIV test	2	0.49%
HIV status	0	0%
Past STI diagnosis		
Syphilis	9	2.22%
Gonorrhea	5	1.23%
Genital warts	10	2.47%
Hepatitis A	11	2.71%
Hepatitis B	13	3.21%
Hepatitis C	13	3.21%
Violence		
Have you ever had sex in exchange for money, help, lodging, protection or gifts?	4	0.99%
Have you ever offered sex in exchange for money, help, lodging, protection or gifts?	2	0.49%
Has a man ever forced you to have sex with him?	6	1.48%
Participation in chemsex parties	0	0%
Sex with HIV-positive men	4	0.99%
Use of drugs to socialize and enjoys sexual practice more		
Alcohol	2	0.49%
Marijuana	3	0.74%
Poppers	7	1.73%
Cocaine	7	1.73%
Ecstasy, MDMA or amphetamines	6	1.48%
LSD	6	1.48%
Viagra, Cialis	7	1.73%
GHB/GBL (liquid ecstasy)	7	1.73%

**Relationships status and meeting place variables**		
Most common type of sexual relationship	11	2.72%
If you have a closed steady partner, this relationship lasted	0	0%
If you have friends with benefits, this relationship lasted	0	0%
Last sexual relationship was	4	0.99%
Venues to meet sexual partners (social media)	0	0%
Badoo	8	1.48%
Grindr	3	0.74%
Manhunt	0	0%
Growler	2	0.49%
Contactossex	5	1.23%
Tinder	1	0.25%
Facebook	6	1.48%
Gay chats	1	0.25%
Meeting places		
Gay bar	3	0.74%
Bar	0	0%
Sex shops	3	0.74%
Disco bar	3	0.74%
Sauna	1	0.25
Cruising	2	0.49%
Public toilets	0	0%
Sex parties	3	0.74%
